# The RANK–RANKL–OPG System: A Multifaceted Regulator of Homeostasis, Immunity, and Cancer

**DOI:** 10.3390/medicina59101752

**Published:** 2023-09-30

**Authors:** Diego De Leon-Oliva, Silvestra Barrena-Blázquez, Laura Jiménez-Álvarez, Oscar Fraile-Martinez, Cielo García-Montero, Laura López-González, Diego Torres-Carranza, Luis M. García-Puente, Sara T. Carranza, Miguel Ángel Álvarez-Mon, Melchor Álvarez-Mon, Raul Diaz, Miguel A. Ortega

**Affiliations:** 1 Department of Medicine and Medical Specialties, Faculty of Medicine and Health Sciences, University of Alcalá, 28801 Alcala de Henares, Spain; diego.leon@edu.uah.es (D.D.L.-O.); silvebarrena@gmail.com (S.B.-B.); laura.jimenezal@gmail.com (L.J.-Á.); oscarfra.7@hotmail.com (O.F.-M.); cielo.gmontero@gmail.com (C.G.-M.); diegotc90@gmail.com (D.T.-C.); lmgarciapuente@gmail.com (L.M.G.-P.); storrescarranza@gmail.com (S.T.C.); maalvarezdemon@icloud.com (M.Á.Á.-M.); mademons@gmail.com (M.Á.-M.); 2 Ramón y Cajal Institute of Sanitary Research (IRYCIS), 28034 Madrid, Spain; 3 Department of Nursing and Physiotherapy, Faculty of Medicine and Health Sciences, University of Alcalá, 28801 Alcala de Henares, Spain; 4 Surgery Service, University Hospital Principe de Asturias, 28801 Alcala de Henares, Spain; 5 Department of Surgery, Medical and Social Sciences, Faculty of Medicine and Health Sciences, University of Alcalá, 28801 Alcala de Henares, Spain; laura.lgonzalez@uah.es; 6 Immune System Diseases-Rheumatology Service, University Hospital Principe de Asturias, 28801 Alcala de Henares, Spain

**Keywords:** RANKL, RANK, osteoclast, immune system, bone metastasis, breast cancer

## Abstract

The RANK–RANKL–OPG system is a complex signaling pathway that plays a critical role in bone metabolism, mammary epithelial cell development, immune function, and cancer. RANKL is a ligand that binds to RANK, a receptor expressed on osteoclasts, dendritic cells, T cells, and other cells. RANKL signaling promotes osteoclast differentiation and activation, which leads to bone resorption. OPG is a decoy receptor that binds to RANKL and inhibits its signaling. In cancer cells, RANKL expression is often increased, which can lead to increased bone resorption and the development of bone metastases. RANKL-neutralizing antibodies, such as denosumab, have been shown to be effective in the treatment of skeletal-related events, including osteoporosis or bone metastases, and cancer. This review will provide a comprehensive overview of the functions of the RANK–RANKL–OPG system in bone metabolism, mammary epithelial cells, immune function, and cancer, together with the potential therapeutic implications of the RANK–RANKL pathway for cancer management.

## 1. Introduction to the RANK–RANKL–OPG System

During the late 1990s, the RANK–RANKL–OPG system was elucidated as a pivotal regulator of bone remodeling [1,2]. Subsequently, its multifaceted role has unfolded, encompassing significant contributions to immune function modulation, lymph node and thymus organogenesis, mammary gland development during pregnancy, thermoregulation, and the control of fever or hair growth [3,4,5,6,7,8]. The expression of RANK and RANKL has also been closely linked to cancer, with more extensive research having been conducted in the context of bone metastasis and breast cancer. In addition, special attention has been paid to elucidating their role in multiple myeloma, urological malignancies, and lung carcinoma [9,10,11].

The RANK–RANKL–OPG system is composed of three members that interact with each other. The receptor activator of the nuclear factor-κB ligand (RANKL; also known as TNFSF11, ODF, OPGL or TRANCE) is a member of the tumor necrosis factor-α (TNF-α) superfamily. It binds to and activates the signaling receptor activator of nuclear factor-κB (RANK; also known as TNFRSF11A, ODFR, or TRANCER). Classically, the RANKL stimulation was described as an osteoclast differentiation promoter, contributing to bone resorption (see Section 3.1) and increased dendritic cell-stimulated naïve T cell proliferation and survival (see Section 3.3.2) [12]. Osteoprotegerin (OPG; also known as TNFRSF11B) is another member of the TNF receptor superfamily and acts as a decoy receptor that interacts with both membrane and soluble RANKL, inhibiting the activation of RANK signaling and thereby limiting osteoclast formation [1,13]. These discoveries of the involvement of RANK–RANKL–OPG in osteoclastogenesis and immunomodulation led to the development of the field of osteoimmunology [14]. Due to the relevance of the RANK–RANKL signaling pathway, the RANKL-neutralizing antibody denosumab has been developed for the treatment of postmenopausal osteoporosis, bone metastases, and skeletal-related events (SRE) [15].

In this review, we aim to comprehensively summarize the functions of the RANK–RANKL–OPG system in bone metabolism, mammary epithelial cells, and the immune system to better understand the intricate interplay between RANK, RANKL, and cancer, highlighting their contribution to bone metastasis, breast and other cancers. In addition, we delve into the potential therapeutic implications of the RANK–RANKL pathway for cancer management.

## 2. The RANK–RANKL–OPG System

RANK is the signaling receptor of RANKL. It consists of a type I transmembrane protein of 616 aa. It presents four cysteine-rich domains (CRD) in the extracellular N-terminus, typical of the TNF receptor superfamily [16]. The long intracellular C-terminus contains TNFR-associated factor (TRAF)-binding motifs. RANK is expressed by osteoclasts, dendritic cells (DCs), T cells, mammary epithelial cells, and medullary thymic epithelial cells [17]. The binding of RANKL and RANK by cell contact or soluble RANK leads to the clustering of RANK and the activation of downstream signaling through the recruitment of TRAFs to the cytoplasmic tail of RANK. The RANK signaling pathways include NF-κB, mitogen-activated protein kinases (MAPKs), PI3K/Akt/mTOR, and NFATc1, and these control the transcription of numerous effector genes [18,19].

The gene TNFSF11 encodes for RANKL a 317 aa type II transmembrane protein with a C-terminal extracellular domain [20]. It is expressed by both T and B lymphocytes, mammary epithelial cells, osteoblasts, osteocytes and bone marrow stromal cells, among others [21]. The soluble form of RANKL arises from a proteolytic cleavage by A disintegrin and metalloproteinase domain-containing protein 10 (ADAM10), or matrix metalloproteinase-14 (MMP-14), or an alternative splicing [22]. The soluble form contributes more significantly to bone remodeling in adult mice [23]. Both the membrane and the soluble isoforms assemble into homotrimers, a characteristic feature of the TNF family, and are biologically active [16].

Osteoprotegerin (OPG; also known as TNFRSF11B) is another member of the TNF receptor superfamily. It acts as a decoy receptor that interacts with both membrane and soluble RANKL, inhibiting the activation of RANK signaling, and thereby limiting osteoclast formation [1,13]. OPG hodimerizes before binding to RANKL [24]. Moreover, the OPG–membrane RANKL leads to the internalization of OPG mediated by RANKL via the clathrin pathway to the lysosomal and proteasomal degradation [25]. Therefore, the suppressive impact of OPG on bone resorption can be elucidated not solely by its role as a decoy receptor and a competitive inhibitor of the RANK–RANKL binding, but also through its ability to alter the half-life of RANKL. Conversely, this modulation actively contributes to diminishing the bioavailability of OPG. Recently, another player has been identified, the leucine-rich repeat-containing G-protein-coupled receptor 4 (LGR4; also known as GPR48) [26]. It competes with RANK for the binding of RANKL and activates the Gαq and GSK3-β signaling pathways, suppressing the expression of NFATc1.

## 3. Functions of RANK–RANKL

RANK–RANKL have a wide range of specific functions, which vary according to the tissues where they are expressed.

### 3.1. Osteoclast Differentiation and Activation

**Figure 1:**
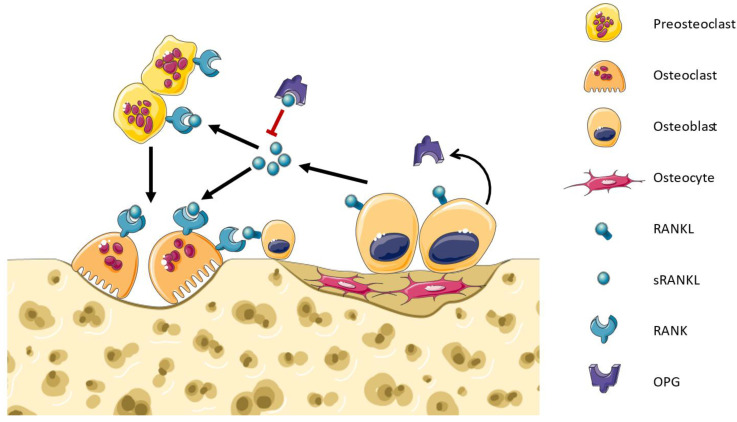
Bone remodeling. Osteoblasts express soluble and membrane-bound RANKL which binds to RANK in the membrane of osteoclast precursors. RANK signaling activates the differentiation towards osteoclasts. RANK signaling in osteoclasts promotes their bone resorptive activity and survival. Osteoblasts infiltrate into the cavities and synthesize the bone matrix. OPG is a decoy receptor produced by osteoblasts that binds RANKL, inhibiting the RANK signaling and regulating bone remodeling.

Bone remodeling is a continuous and dynamic biological process characterized by the concurrent activities of osteoclast-mediated bone resorption and osteoblast-mediated bone formation. This helps maintain bone strength, repair microdamage, and regulate calcium levels in the body [27,28]. The osteoclasts form resorption cavities in the bones that are subsequently filled with osteoblasts that lay down new bone matrices in the walls of these cavities [29]. RANK–RANKL play an important role in bone resorption, as they are responsible for osteoclast differentiation, activity, and survival (Figure 1) [30,31]. RANKL produced by osteocyte signaling induces c-fos, NFATc1/NFAT2, and canonical and non-canonical NF-κB pathways, which upregulate the genes tartrate-resistant acid (TRAP), cathepsin-K, calcitonin receptor, as well as c-myc to promote OC proliferation [32]. A disruption in the equilibrium between RANKL and OPG can result in either osteoporosis or osteopetrosis [33]. Osteocytes produce either soluble or membrane RANKL. The regulatory proteins RANKL, OPG, and sclerostin can also be released into the extracellular medium by the osteocytes within extracellular vesicles (EVs) via a Ca^2+^-dependent mechanotransduction signaling pathway that induces changes in the cytoskeletal arrangement [34]. However, the main mechanism is not fully clear because the osteocytes are embedded in the bone matrices while the osteoclast precursors are localized in the bone marrow cavities [35]. Honma et al. conducted co-cultures of osteocytes and osteoclast precursor cells, employing a pore membrane to physically separate the two cell types. Their findings revealed that the use of small-pore membranes effectively prevented direct contact between these cell populations, leading to a reduction in the efficiency of osteoclast formation [36]. On the other hand, the conditioned media derived from apoptotic MLO-Y4 cells exhibited an enhancement in osteoclast formation, an increase in osteoclast size, and an augmented migration of osteoclast precursors. Notably, within the apoptotic MLO-Y4 cells, both mRNA and the protein expression of RANKL were upregulated, implying that the conditioned media from this cell culture potentially contain soluble RANKL, possibly in EVs [37]. Therefore, certain authors suggest that the delivery mode of osteocytic RANKL may be influenced by specific conditions [38]. Interestingly, recent research has shown that RANKL can act in an opposite sense (reverse signaling), like other TNF superfamily members, and induce osteoblast differentiation and bone formation [39,40]. This demonstrates that our knowledge of bone remodeling remains limited and that further research is still needed for more precise therapies against osteoporosis and SRE.

### 3.2. Formation of Lactating Mammary Glands

**Figure 2:**
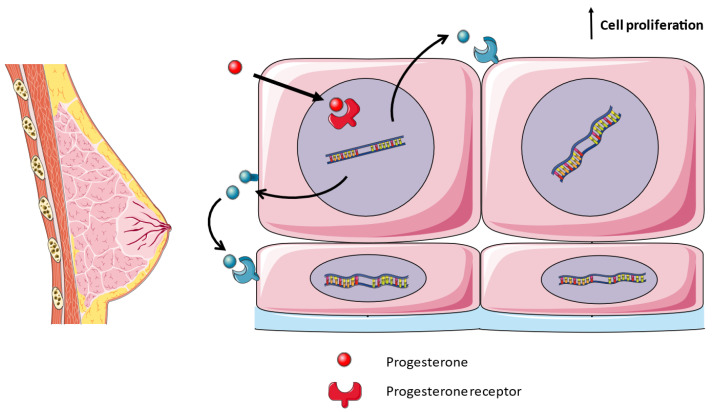
Development of lactating mammary glands. During pregnancy, progesterone binds to its cytoplasmic receptor, upregulating the expression of RANKL in luminal epithelial cells. RANKL binds to RANK in the neighboring cells, either luminal, basal or mammary stem cells, in a paracrine fashion. RANK signaling activates cell proliferation.

During pregnancy, the breasts undergo the finalization of the developmental processes initiated during puberty, facilitating lactation through anatomical and physiological transformations. These changes are primarily orchestrated by the hormonal actions of progesterone and estrogen [41]. Throughout pregnancy, the expansion and proliferation of ductal and alveolar epithelial cells leads to an increase in ductal side branching and the development of lobulo–alveolar structures. RANK–RANKL are essential mediators for the formation of mammary glands in pregnancy. RANK- and RANKL-deficient mice suffer a block in the formation of lobulo–alveolar structures, which produce milk, due to a lack of proliferation of epithelial cells [42]. In women, pregnancy increases the tissue expression of RANKL [43]. Progesterone, acting through its receptor, triggers the expression and secretion of RANKL by luminal epithelial cells. RANKL then engages with RANK in both an autocrine and paracrine fashion, thereby stimulating cellular proliferation, including the pool of mammary stem cells, and the formation of milk-secreting acini throughout pregnancy (Figure 2) [44,45]. RANK signaling during mammary gland development comprises cyclin D1 expression mediated by the IKKα subunit of the IκB kinase and the WNT pathway enhanced by R-SPONDIN1 [46,47]. Moreover, a rise in the serum levels of RANKL and OPG in healthy pregnant women is associated with a greater or smaller increase in breast volume, respectively [48].

### 3.3. Roles in the Immune System

The immune system comprises a complex network of molecules, cells and tissues that work together to defend the body against external (e.g., infections) and internal (e.g., cancer cells) threats. Its responses are classified into two groups: innate, non-specific, and adaptive, highly specific. The RANK–RANKL–OPG system plays multiple roles at different levels in the immune system that, as we are going to review, are of great importance for the development of cancer.

#### 3.3.1. Organogenesis of Lymphoid Organs

First, as we have seen for mammary glands, RANK–RANKL is of great importance for the development of epithelial tissues in various organs. In the case of the immune system, they participate in the development and growth of lymph nodes, the establishment of the thymic and bone marrow microenvironment, and M cell differentiation in the intestinal tract [5,49,50,51]. From the initial observations, it became evident that RANK–RANKL plays a pivotal role in the regulation of lymph node development, as evidenced by the complete absence of these secondary lymphoid organs in mice lacking RANK or RANKL [52]. During embryogenesis, RANKL is expressed by lymphoid tissue-inducing (LTi) cells and lymphoid tissue organizer (LTo) cells which stimulate lymphotoxin (LT) expression and regulate LTi cell accumulation to initiate lymph node organization [53,54]. Regarding the establishment of the thymic microenvironment, RANK signaling is essential for medullary thymic epithelial cell (mTEC) development during embryogenesis, while cooperation between CD40 and RANK signals is required postnatally to establish the medullary microenvironment, crucial for self-tolerance in the immune system, and may also be involved in the thymic involution [5,55,56]. Defects in the RANK–RANKL system can result in the excessive ossification of bone, leading to the obliteration of bone marrow cavities and subsequent alterations in hematopoiesis. Thus, indirectly, RANK–RANKL collaborate on the formation of this organ [50]. Lastly, RANK signaling is necessary for the M cell differentiation in the intestinal epithelium, where RANKL is provided by the stromal cells to the RANK-expressing stem cells [51,57].

#### 3.3.2. Regulation of Immune Cells

RANKL–RANK signaling plays a crucial role in the activation and survival of DCs and T cells. RANK signaling in DCs activates the NF-κB and MAPK pathways, leading to the transcriptional activation of genes required for proliferation, survival, and differentiation [58]. DCs are essential for antigen presentation and T cell activation. RANKL treatment enhances the ability of the DCs to prime the T cells, promoting an immune response. RANKL signaling also regulates the function of T cells, promoting their activation and survival [59]. Both RANK and CD40 (members of the TNF receptor family) activate similar intracellular signaling pathways in DCs. They share binding sites for TRAF family proteins [60]. However, CD40 signaling also upregulates the expression of co-stimulatory molecules and MHC molecules on DCs.

RANKL has been implicated in B cell recruitment and organization in lymphoid tissues. B cell follicular dendritic cells (FDCs) and marginal reticular cells (MRCs) produce the chemokine CXCL13, which is essential for B cell recruitment. Studies have shown that RANKL signaling is involved in the development of FDCs and the production of CXCL13, as evidenced by the absence of B cells and FDCs in mice lacking RANK signaling [53,61]. This suggests that RANKL plays a role in B cell recruitment and follicle organization in lymphoid tissues.

On the macrophages, RANKL inhibits toll-like receptor 4 (TLR4) activation due to the recruitment of TRAF6 to the intracytoplasmic tail of RANK instead of TLR4, lowering the expression of proinflammatory mediators [62].

#### 3.3.3. Other Immune Functions in the Skin, Central Nervous, and Skeletal Systems

In the skin, RANKL is expressed by keratinocytes exposed to ultraviolet and engages with RANK in the Langerhans cells, leading to the proliferation of regulatory T (Treg) cells, mediating immunosuppressive effects [63]. In the context of the central nervous system (CNS), divergent roles have been observed for the RANK–RANKL axis. On the one hand, in models of multiple sclerosis, it has been demonstrated that Th17 cells expressing RANKL engage with RANK on astrocytes. This interaction subsequently triggers the secretion of the C–C motif chemokine ligand 20 (CCL20), which, in turn, recruits cells expressing the C–C motif chemokine receptor 6 (CCR6), including Th17 cells, into the CNS [64]. On the other hand, in the context of ischemic stroke, the RANK–RANKL signaling pathway exerts an inhibitory effect on the production of proinflammatory cytokines by microglial cells [65]. In a retrospective study, the rs9533156 gene polymorphism of the RANKL gene was associated with history of ischemic stroke [66]. Other studies have revealed an association between elevated plasma OPG levels and poor functional outcomes measured using the modified Rankin scale in acute ischemic stroke patients [67,68].

Osteoimmunology is a field of research that studies the interactions between the skeletal and the immune systems, essential for the understanding of bone-related diseases such as osteoporosis or rheumatoid arthritis. It was born upon the discovery that RANKL is a key mediator of the crosstalk between immune cells and osteoclasts [69]. It primarily regulates osteoclast differentiation and formation. Moreover, the immune system modulates osteoclast activity through the production of RANKL and cytokines like IL-1 [14]. Beyond bone regulation, RANKL exhibits immunosuppressive effects by promoting Treg cell generation and influencing dendritic cell activation and survival [70]. Notably, co-stimulatory molecules originally identified in the immune system, such as FcRγ and DAP12, play vital roles in RANK expression and osteoclastogenesis, highlighting the intricate crosstalk between these systems in osteoimmunology [71]. Many diseases are behind the pathologic relationships between immune and bone cells: rheumatoid arthritis, periodontal disease, osteoporosis, osteoarthritis, multiple myeloma, and metastatic bone tumors [72]. The best studied example is rheumatoid arthritis (RA), a chronic disease characterized by its ability to induce inflammation and cartilage and bone destruction. Its precise etiology remains unknown, but it is believed to be mediated by immune mechanisms [73]. Animal models of RA lacking RANKL expression demonstrate joint inflammation without the development of focal bone loss [74]. The secretion of IL-17 by Th17 cells stimulates synovial fibroblasts under inflammatory conditions to induce the expression of RANKL, thereby promoting osteoclastogenesis and subsequent bone destruction [75]. However, Th17 cells that lose Foxp3 expression and knockout for IL-17 can induce osteoclastogenesis on their own through the expression of RANKL [76]. Recent evidence points to a proinflammatory role for RANKL in modeled RA. Papadaki et al. conducted research demonstrating that the simultaneous upregulation of RANKL and TNF in double transgenic mice accelerated the disease onset, leading to the development of severe arthritis [77]. This was characterized by a significant increase in both clinical and histological scores, with clear indications of aggressive pannus formation, extensive bone resorption, and a substantial accumulation of inflammatory cells, primarily of myeloid lineage. Furthermore, it is noteworthy that TNF overexpression can induce osteoclastogenesis independently of RANKL at sites of inflammatory infiltration, and the combined action of TNF and RANKL operates systematically, even affecting distal femurs [77]. Hence, based on the clinical data, denosumab is shown to have advantageous effects in the prevention of bone destruction while having a limited impact on joint inflammation. Therefore, it is advisable to consider its use in conjunction with other drugs, such as methotrexate or biologics, for the comprehensive management of rheumatoid arthritis [78].

## 4. RANK–RANKL and Tumor Growth

### 4.1. Bone Metastasis

The bones, together with the liver and the lungs, are the most common site of metastases. Bone metastases (BM) arise from diverse cancers, e.g.,: breast, prostate or lung [79]. A key event in the progression of bone metastases is the interaction between tumor cell receptors and stromal cells present in the bone marrow and bone matrix [80]. In this context, the RANK–RANKL–OPG system acquires a significant importance in bone metastasis. It facilitates cancer cell migration and its regulatory role in bone remodeling is instrumental in creating a favorable microenvironment, often referred to as “soil”, for the infiltration of metastatic tumor cells [18]. The expression of RANK by some metastatic cancer cells of epithelial tumors leads to the attachment of RANKL in the bones [81]. Furthermore, cancer cells can directly express RANKL or secrete a parathyroid hormone-related protein (PTHrP) to enhance the secretion of RANKL, leading to osteolysis [9,82]. Insulin-like growth factor (IGF), fibroblast growth factor (FGFs), platelet-derived growth factor (PDGF), bone morphogenetic proteins (BMPs) and transforming growth factor beta (TGF-β) are liberated from the bone matrix and promote cancer cell proliferation [83]. Additionally, tumor cells can release various cytokines, including but not limited to interleukin (IL)-1α, IL-6, IL-8, IL-11, TNF-α, macrophage colony-stimulating factor (M-CSF), or prostaglandin E2 (PGE2), which also contribute to osteoclast activation [84]. These establish a “vicious cycle” between the distinct cellular populations that promote osteolysis, liberation of growth factors, and tumor growth.

### 4.2. Multiple Myeloma

Multiple myeloma is a malignant proliferation of monoclonal plasma cells within the bone marrow that results in bone lesions due to osteolysis, caused by the increased activity of osteoclasts [85]. The RANK–RANKL pathway plays a significant role in the pathogenesis of multiple myeloma and is the target of current therapies, such as denosumab. In the 5T2MM mouse model, the myeloma cells express RANKL, directly promoting osteolysis [86]. It seems that RANKL is upregulated during the progression of multiple myeloma [87]. Moreover, serum concentrations of RANKL and the ratio of RANKL–OPG have exhibited notable correlations with angiogenesis markers, such as VEGF, in individuals diagnosed with multiple myeloma [88].

### 4.3. Breast Cancer

Breast cancer is the most common female cancer, affecting approximately one in eight women during their lifetime. As we have reviewed above, the RANK–RANKL system is involved in the development of the mammary glands during pregnancy. It regulates the proliferation of mammary epithelial cells. Therefore, imbalances in the RANK–RANKL pathway play a role in the occurrence of breast cancer.

First, as well as in other types of cancer, the expression of RANK–RANKL is directly correlated with the proliferation and survival of mammary epithelial cells, contributing to the initiation and progression of breast cancer [89]. The role of progesterone is critical in breast cancer. Progesterone and its synthetic derivatives, known as progestins, find widespread use in combined hormone replacement therapy (HRT), as well as in various forms of hormonal contraception. Progestins are linked to an increased risk of breast cancer [90]. It seems that the upregulation of RANK–RANKL in the mammary tissues by progesterone drives the excessive proliferation of mammary epithelial and mammary stem cells in breast cancer [91].

The majority of inherited breast cancers (5–10% of cases) are caused by mutations in the tumor-suppressor genes, breast cancer 1/2 (*BRCA1/2*). On the one hand, BRCA1 and 2 suppress tumorigenesis by promoting the repairing of double-strand breaks, which maintains genomic integrity [92]. On the other hand, sex hormones are involved in the development of breast cancer associated with BRCA1 mutations [93]. For this reason, several studies have shown RANK and RANKL’s role in linking the sex hormones and the proliferation of mammary progenitor cells to the development ofBRCA1-mutated breast cancers [4,94].

Lastly, the RANK–RANKL pathway is also involved in the induction of epithelial-to-mesenchymal transition (EMT), which contributes to invasion, metastasis and resistance to cancer treatment [95]. Palafox et al. found that RANK overexpression induces EMT in non-transformed human mammary epithelial cells [96]. According to Tsubaki et al., RANK signaling activates the NF-κB pathway and upregulates snail and twist, which are two repressors of E-cadherin [97]. RANK–RANKL also contribute to metastasis by inducing senescence and stemness in mammary epithelial cells and by tumor-infiltrated Tregs, which produce RANKL [98,99].

### 4.4. Urologic Cancers

Prostate cancer cells overexpress RANK–RANKL, which is correlated with more aggressive and metastatic carcinoma, suggesting a role for a prognostic biomarker [100,101]. RANK–RANKL together with MMP-1 were demonstrated to promote the metastatic phenotype in prostate cancer cells [102]. Regarding renal cell carcinoma (RCC), RANKL expression was associated with the stimulation of cancer cell migration and metastasis to bones, skin and liver [103]. Lastly, bladder cancer cells also overexpress RANKL [104]. Indeed, Oncotherad immunotherapy associated with platelet-rich plasma (PRP) stimulates an effective immune response by decreasing RANK–RANKL expression and the number of Tregs [105]. However, their role in metastasis remains unclear.

### 4.5. Lung Cancer

Lung cancer cells also express RANK and RANKL, induced by KRas [106]. High-RANK pathway activity has been correlated with decreased survival rates in patients with lung cancer, and its inhibition using the drug denosumab can reduce the growth of lung cancer tumors [107]. Indeed, RANK reconfigures mitochondrial bioenergetics in lung cancer cells and promotes the expansion of cancer stem-like cells. Lastly, it becomes evident that the activation of RANK by female sex hormones may contribute to the acceleration of lung cancer development [108]. This phenomenon provides a potential explanation for the sex and gender differences observed in epidemiological studies of lung cancer [107]. Moreover, the expression of RANK, RANKL and OPG is correlated with the metastatic potential of non-small cell lung cancer (NSCLC) cells [109]. Indeed, the available clinical data regarding the use of denosumab strongly support the involvement of the RANK–RANKL axis in the carcinogenesis of lung cancer. In an observational study, it was observed that patients with metastatic lung cancer exhibited superior overall survival when compared to those treated with zolendronic acid [110].

## 5. Therapeutic Implications in Cancer

Given the crucial pathophysiological role of the RANK–RANKL–OPG system in the body, it has emerged as an intriguing therapeutic target. The initial approach involved the development of a fusion protein known as OPG-Fc, which specifically targets and inhibits RANKL. OPG-Fc (AMGN-0007) comprises the ligand-binding domain of recombinant OPG (residues 22-194) fused with the IgG Fc region [111]. This innovative therapy underwent preclinical investigations and phase I clinical trials, demonstrating promising antiresorptive effects as anticipated. However, the clinical use of OPG-Fc faced challenges. OPG possesses the ability to bind to multiple ligands beyond RANKL, notably the TNF-related apoptosis-inducing ligand (TRAIL). Additionally, concerns arose regarding the potential for a neutralizing immune response against endogenous OPG. As a result, the utilization of OPG-Fc was suspended [112]. Currently, OPG-Fc is employed in murine models to mimic denosumab.

To overcome the disadvantages of a fusion protein, an anti-RANKL antibody was developed, denosumab (first known as AMG 162), which blocks the RANK–RANKL binding. It is a fully human monoclonal IgG2κ antibody that neutralizes with high affinity and specificity for RANKL [113]. It has a moderately lower affinity than OPG-Fc, which is fully compensated by its significantly longer half-life in circulation, which allows reduced dosing frequency and binds both the soluble and the membrane forms of RANKL [114]. Denosumab exhibits primate-specific activity, while OPG-Fc is utilized in murine models due to their highly similar mechanisms of action. Denosumab effectively inhibits bone resorption and cancer-induced bone destruction by competitively binding RANKL and blocking the differentiation, activation, and survival of osteoclasts [115]. Therefore, denosumab is indicated and FDA-approved for the following: postmenopausal and glucocorticoid-induced osteoporosis, androgen deprivation-induced and aromatase inhibitor-induced bone loss, prevention of SRE secondary to multiple myeloma or bone metastasis, giant cell tumors, and hypercalcemia of malignancy [15,116].

The antitumor effects of denosumab have been described as either indirect or direct. The former implies the RANKL inhibition of osteoclasts leading to the disruption of the “vicious cycle”. The release of growth factors is reduced and the bone microenvironments become less attractive for tumor cells as a site for growth and metastasis [117]. It also inhibits angiogenesis. The latter involves the RANK signaling inhibition of the tumor cells, which may lead to the promotion of tumor cell apoptosis and decrease migration [118].

Bortezomib, a proteasome inhibitor, effectively restores normal bone resorption in individuals with multiple myeloma. This therapeutic agent has been demonstrated to reduce serum RANKL levels [119]. In patients with multiple myeloma undergoing autologous stem cell transplantation, a decline in the soluble RANKL/OPG ratio and bone resorption markers is observed initially [120]. Subsequently, at the 9th and 11th months, the markers of bone formation commence to rise.

The prognosis in various cancer types, such as breast, lung, endometrial, renal cell, gastric cancers, osteosarcoma, and multiple myeloma, can be affected by the relative expression levels of RANK, RANKL, and OPG [121]. Pfitzner et al. analyzed the tissue expression of 601 patients with breast cancer, the disease best studied, and found that high RANK expression was associated with high pathological complete response, shorter disease-free and overall survival, and higher sensitivity to chemotherapy, but also a higher risk of relapse and death [10]. Ciscar et al. showed that RANK expression in estrogen receptor-negative breast cancer was associated with poor outcomes and poor responses to chemotherapy [122]. Also, patients with the single nucleotide polymorphism rs34945627 had decreased disease-free and overall survival [123].

## 6. Conclusions

The RANK–RANKL–OPG system, initially recognized as a fundamental regulator of bone remodeling through its influence on osteoclast formation and activation, has since revealed a multitude of significant functions across diverse biological processes. These include the regulation of the immune processes, the development of the lymphoid organs, and the facilitation of lactation in the mammary glands. The multifaceted functions of this system have implications throughout the various stages of tumorigenesis, with notable relevance to conditions such as bone metastases, multiple myeloma, breast cancer, urological cancers and lung cancer. The clinical approval of denosumab, an anti-RANKL neutralizing antibody, for the treatment of osteoporosis and cancer-induced bone loss underscores the therapeutic potential of targeting this pathway in diverse medical contexts. However, it is essential to emphasize that our comprehension of the intricate biology of RANK and RANKL remains incomplete. Therefore, further research is imperative to delve deeper into the mechanisms and regulation of this system. Such efforts are not only critical to advance our knowledge, but also hold the promise of refining existing therapeutic approaches, which could lead to innovative treatments with greater efficacy and fewer side effects. In this context, the ongoing exploration of the RANK–RANKL–OPG system promises to be fertile ground for future scientific research and clinical advances.

## Data Availability

Not applicable.
